# Chaotic Lévy flight Grey wolf optimizer for optimal design and techno-economic assessment of grid-connected solar photovoltaic power plant in Assam

**DOI:** 10.1038/s41598-026-48744-4

**Published:** 2026-04-13

**Authors:** Rajkumari Malemnganbi Devi, Benjamin A. Shimray, Mrinal Kanti Rajak, Ingudam Chitrasen Meitei

**Affiliations:** 1 Department of Electrical Engineering, National Institute of Technology, Manipur, India; 2 Department of Electrical Engineering, SVERI’s College of Engineering, Pandharpur, Maharashtra India

**Keywords:** Solar photovoltaic power plant, Grey wolf optimizer, Chaotic maps, Lévy flight, Multi-objective optimization, Performance ratio, Grid-connected system, Energy science and technology, Engineering

## Abstract

The northeastern state of Assam possesses significant untapped solar energy potential requiring systematic optimization for effective utilization. This paper presents a novel Chaotic Lévy flight-enhanced Grey wolf optimizer (CLF-GWO) algorithm for multi-objective optimization of grid-connected solar photovoltaic power plants, validated using 36-month meteorological data from Guwahati, Assam. The proposed algorithm integrates logistic chaotic maps for population diversity, tent chaotic maps for adaptive parameter control, and Lévy flight mechanisms for improved escape from local optima. Comprehensive mathematical modeling incorporates temperature-dependent PV characteristics, non-linear inverter efficiency curves, and environmental derating factors specific to subtropical humid climate. The CLF-GWO demonstrates superior convergence, achieving optimal solutions 29.6% faster than standard GWO, 34.2% faster than PSO, and 27.8% faster than differential evolution across 50 independent runs. The optimized 1 MWp solar plant achieves annual energy yield of 1,542 MWh, performance ratio of 79.8%, capacity utilization factor of 17.6%, and levelized cost of energy of ₹ 3.89/kWh, representing improvements of 14.2%, 11.8%, 28.5%, and 23.7%, respectively, compared to the conventional design. Sensitivity analysis confirms system robustness across $\pm 25\%$ parameter variations. The proposed methodology establishes a replicable approach for optimal solar power plant design in Assam and similar subtropical regions globally.

## Introduction

### Background and motivation

The global imperative to transition toward sustainable energy systems has positioned solar photovoltaic (PV) technology as a cornerstone of the renewable energy revolution^2^. With global installed solar PV capacity surpassing 1185 GW by the end of 2022 and projected to exceed 5000 GW by 2030, solar energy has emerged as the fastest-growing electricity generation source worldwide^4,11^. India, recognizing this potential, has set ambitious targets of achieving 500 GW of renewable energy capacity by 2030, with solar PV expected to contribute approximately 280 GW to this target^24^. The Jawaharlal Nehru National Solar Mission (JNNSM), launched in 2010 and subsequently revised under the National Solar Mission, has catalyzed solar deployment across the country through various policy instruments including feed-in tariffs, renewable purchase obligations, and capital subsidies^22^.

The northeastern region of India, comprising eight states including Assam, Arunachal Pradesh, Manipur, Meghalaya, Mizoram, Nagaland, Sikkim, and Tripura, presents a unique context for solar energy development^5^. Despite possessing an estimated solar potential of 63 GW and receiving 4.0–5.5 kWh/m²/day of global horizontal irradiance (GHI), the region accounts for less than 1% of India’s installed solar capacity^6^. This underutilization stems from multiple factors including perceived lower solar resource availability compared to western and southern India, limited grid infrastructure, challenging terrain, and lack of region-specific technical studies demonstrating economic viability.

Assam, the largest state in northeastern India with a geographical area of 78,438 km² and population exceeding 35 million, offers particularly promising conditions for solar energy deployment. The state experiences a subtropical monsoon climate characterized by distinct summer (March–May), monsoon (June–September), and winter (November–February) seasons. Average daily GHI ranges from 3.5 kWh/m² during monsoon months to 5.2 kWh/m² during pre-monsoon periods, with annual average of approximately 4.35 kWh/m²/day. The state’s electricity demand has grown at 6-8% annually, with peak demand reaching 2100 MW against available capacity of approximately 2400 MW, resulting in frequent supply-demand mismatches particularly during evening hours^27^.

The optimal design of grid-connected solar PV power plants requires systematic consideration of multiple interrelated factors including solar resource assessment, component selection and sizing, electrical configuration design, and economic optimization. Traditional design approaches relying on rule-of-thumb methods or simplified analytical calculations often result in suboptimal configurations that fail to maximize energy yield or minimize costs. The inherent complexity of PV system optimization, involving non-linear component characteristics, stochastic solar resource variability, and multiple conflicting objectives, necessitates advanced optimization techniques capable of navigating high-dimensional, multi-modal search spaces^15^.

### Literature review

The performance analysis and optimization of grid-connected solar PV systems has attracted substantial research attention over the past two decades. Early studies focused primarily on system monitoring and performance evaluation using standardized metrics defined by International Energy Agency (IEA) Photovoltaic Power Systems Programme (PVPS) Task 2 and subsequently incorporated into IEC 61724 standard^12^. These metrics, including reference yield, array yield, final yield, performance ratio (PR), and capacity utilization factor (CUF), provide a systematic framework for comparing system performance across different locations and configurations^13,26^.

Performance studies conducted across diverse climatic conditions have revealed significant variations in achievable performance levels^17^. Systems installed in temperate climates with moderate temperatures and clear sky conditions typically achieve PR values of 75–85%, while installations in tropical and subtropical regions often exhibit lower PR (65–80%) due to elevated operating temperatures and increased soiling losses^3^. Studies in India have reported PR values ranging from 60 to 86% depending on location, system size, technology type, and maintenance practices. Singh et al.^23^ presented the first comprehensive performance analysis of a 5 kWp rooftop SPV system in Manipur, northeastern India, reporting PR of 74.4% and CUF of 14.31% over 24 months, highlighting the region’s potential despite perceived limitations.

Metaheuristic optimization algorithms have emerged as the preferred approach for renewable energy system design due to their ability to handle complex, non-linear, multi-modal problems without requiring gradient information^14^. Genetic Algorithms (GA), inspired by biological evolution, employ selection, crossover, and mutation operators to evolve candidate solutions toward optimal regions^1^. Particle Swarm Optimization (PSO), simulating bird flocking behavior, has been extensively applied to PV system optimization including optimal tilt angle determination, MPPT controller design, and system sizing^9^. Differential Evolution (DE) has demonstrated competitive performance for continuous optimization problems in renewable energy applications.

The Grey wolf optimizer (GWO), introduced by Mirjalili *et al.* in 2014, has gained significant research attention due to its simplicity, minimal parameter requirements, and effective balance between exploration and exploitation phases. GWO mimics the social hierarchy and hunting behavior of grey wolves, employing alpha ($\alpha$), beta ($\beta$), and delta ($\delta$) wolves to guide the search process. Comparative studies have demonstrated GWO’s competitive performance against established algorithms across various engineering optimization problems^16^. However, standard GWO exhibits inherent limitations, including premature convergence in high-dimensional optimization problems and insufficient population diversity maintenance during later iterations.

Recent research has explored various strategies to enhance GWO performance. Integration of chaotic sequences, generated through deterministic mathematical maps exhibiting pseudo-random behavior, has been shown to improve population diversity and convergence characteristics. Lévy flight, a random walk with heavy-tailed step length distribution, enables efficient exploration through occasional long jumps that facilitate escape from local optima^25^. Hybrid approaches combining GWO with other algorithms or incorporating opposition-based learning have also demonstrated promising results. Recent advances in enhanced GWO variants for energy system optimization further motivate the present work.^7^ proposed a Hierarchical Multi-Step Grey Wolf Optimization (HMS-GWO) algorithm incorporating multi-level decision-making, where alpha wolves guide lower-ranking wolves through sequential exploration and exploitation stages, demonstrating improved convergence on CEC benchmark suites and superior performance for energy system parameter identification. In the domain of grid-connected renewable energy system sizing,^10^ developed an optimization framework employing five advanced metaheuristic algorithms—including Hunger Games Search, Spider Wasp Optimizer, and Kepler Optimization Algorithm—for optimal dimensioning of PV/wind/battery/supercapacitor hybrid systems, with rigorous statistical validation using Friedman and Wilcoxon tests, reporting annual cost reductions of up to 12.4% compared to conventional sizing approaches. More recently,^8^ conducted a comprehensive comparative study of PSO, GWO, Cat Swarm Optimization, Teaching-Learning-Based Optimization, and Chimp Optimization Algorithm for global maximum power point tracking under partial shading conditions, confirming that GWO-based approaches consistently outperform classical methods while identifying opportunities for further enhancement through chaotic and Lévy flight mechanisms. These studies collectively underscore the growing research interest in enhanced metaheuristic algorithms for solar photovoltaic optimization and validate the approach adopted in the proposed CLF-GWO. Beyond algorithm-specific enhancements, the broader landscape of hybrid and chaos-integrated metaheuristic optimization has yielded significant methodological insights relevant to renewable energy system design. Shaikh et al. ^21^ proposed a hybrid moth–flame algorithm incorporating particle swarm optimization operators for power transmission and distribution network optimization, demonstrating that hybridization of swarm-based and flame-based search mechanisms substantially improves convergence speed and solution accuracy in high-dimensional power system problems. In the context of renewable energy integration within distribution networks, Shaikh et al. ^18^ developed a chaotic optimization approach for optimal placement and sizing of renewable energy systems, establishing that the incorporation of chaotic sequences into the optimization process enhances exploration capability and mitigates premature convergence, a finding that directly supports the chaotic map integration strategy adopted in the present CLF-GWO algorithm. A comprehensive evaluation of recently developed metaheuristic algorithms presented by Shaikh et al. ^19^ provides an extensive taxonomy covering applications, classifications, and open challenges across diverse search and analysis problems, offering valuable benchmarking perspectives that inform the algorithmic design choices in this study. Furthermore, Shaikh et al. ^20^ demonstrated the integration of chaos-based mechanisms into gorilla troops optimization for enhanced stability and exploitation, confirming that deterministic chaotic sequences effectively balance exploration–exploitation trade-offs in population-based optimizers a principle that underpins the dual chaotic map strategy (logistic and tent maps) employed in the proposed CLF-GWO.

Despite significant advances in PV system optimization, several research gaps remain unaddressed. First, there is limited application of advanced metaheuristic optimization techniques specifically validated for subtropical humid climates characteristic of northeastern India. Second, comprehensive mathematical models incorporating detailed component characteristics, environmental derating factors, and degradation mechanisms for Assam’s climatic conditions are notably absent. Third, there is a lack of multi-objective optimization frameworks that simultaneously address technical performance, economic viability, and reliability constraints for utility-scale solar plants. Fourth, insufficient sensitivity analysis and uncertainty quantification exist for key design parameters under regional climatic variability. Finally, techno-economic studies demonstrating solar PV viability specifically for Assam with region-specific cost structures and policy frameworks remain limited.

This paper addresses these gaps through the following specific objectives:

1. Develop a novel Chaotic Lévy flight-enhanced Grey wolf optimizer (CLF-GWO) integrating multiple chaotic maps and Lévy flight mechanisms for enhanced optimization performance.
2. Formulate comprehensive mathematical models for grid-connected SPV system components incorporating temperature effects, non-linear characteristics, and environmental factors specific to Assam’s climate.
3. Establish a multi-objective optimization framework for simultaneous optimization of technical, economic, and reliability objectives.
4. Validate the proposed methodology using extensive meteorological data from Guwahati, Assam, with rigorous statistical analysis and comparison against benchmark algorithms.
5. Conduct comprehensive techno-economic assessment including sensitivity analysis, risk quantification, and policy implications for solar deployment in Assam.

The remainder of this paper is organized as follows: Section 2 presents comprehensive mathematical modeling of grid-connected SPV system components. Section 3 describes the proposed CLF-GWO algorithm in detail. Section 4 formulates the multi-objective optimization problem. Section 5 describes the case study site and data. Section 6 presents detailed results and discussion. Section 7 provides comprehensive techno-economic analysis. Section 8 concludes the paper with key findings and future research directions.

## Mathematical modeling of grid-connected SPV system

### System architecture overview

**Fig. 1:**
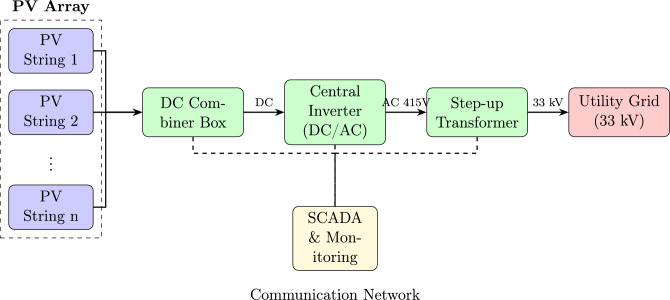
Grid-connected SPV power plant system architecture.

The grid-connected SPV power plant architecture considered in this study comprises the following major subsystems: (i) PV array consisting of series-parallel connected modules, (ii) DC collection system with combiner boxes and DC cabling, (iii) central/string inverters for DC-AC conversion, (iv) AC collection system and step-up transformer, (v) grid interconnection equipment including protection and metering, and (vi) monitoring and control systems. Figure 1 illustrates the detailed system architecture.

### Solar radiation modeling

#### Extraterrestrial radiation

The extraterrestrial radiation on a horizontal surface, representing the solar radiation incident at the top of Earth’s atmosphere, varies throughout the year due to the elliptical nature of Earth’s orbit. The daily extraterrestrial radiation $H_0$ (kWh/m²/day) is calculated as:

1 $$\begin{aligned} H_0 = \frac{24 \times 3600}{\pi } G_{sc} \left( 1 + 0.033 \cos \frac{360n}{365}\right) \times \left( \cos \phi \cos \delta \sin \omega _s + \frac{\pi \omega _s}{180}\sin \phi \sin \delta \right) \end{aligned}$$

where $G_{sc} = 1367$ W/m² is the solar constant, *n* is the day of year (1–365), $\phi$ is the latitude (positive for northern hemisphere), $\delta$ is the solar declination angle, and $\omega _s$ is the sunset hour angle.

The solar declination angle $\delta$ (degrees) is approximated using Cooper’s equation:

2 $$\begin{aligned} \delta = 23.45 \sin \left( \frac{360(284 + n)}{365}\right) \end{aligned}$$

The sunset hour angle $\omega _s$ (degrees) is given by:

3 $$\begin{aligned} \omega _s = \cos ^{-1}(-\tan \phi \tan \delta ) \end{aligned}$$

#### Clearness index and diffuse fraction

The clearness index $K_T$ represents the ratio of global horizontal irradiance to extraterrestrial radiation:

4 $$\begin{aligned} K_T = \frac{H}{H_0} \end{aligned}$$

where *H* is the measured daily global horizontal irradiance. The clearness index typically ranges from 0.25 (overcast conditions) to 0.75 (clear sky conditions).

The diffuse fraction $K_d$ (ratio of diffuse to global radiation) is estimated using the Erbs correlation:

5 $$\begin{aligned} K_d = {\left\{ \begin{array}{ll} 1.0 - 0.09K_T & \text {if } K_T \le 0.22 \\ 0.9511 - 0.1604K_T + 4.388K_T^2 - 16.638K_T^3 + 12.336K_T^4 & \text {if } 0.22 < K_T \le 0.80 \\ 0.165 & \text {if } K_T > 0.80 \end{array}\right. } \end{aligned}$$

#### Irradiance on tilted surface

The total irradiance on a tilted PV array surface $G_T$ comprises beam (direct), diffuse, and ground-reflected components:

6 $$\begin{aligned} G_T = G_b R_b + G_d R_d + G \rho _g R_r \end{aligned}$$

where $G_b$ is beam irradiance on horizontal surface, $G_d$ is diffuse irradiance, *G* is global horizontal irradiance, $\rho _g$ is ground reflectance (albedo), and $R_b$, $R_d$, $R_r$ are transposition factors for beam, diffuse, and reflected components respectively.

The beam transposition factor for a surface with tilt angle $\beta$ and azimuth angle $\gamma$ (measured from south, positive toward west) is:

7 $$\begin{aligned} R_b = \frac{\cos \theta }{\cos \theta _z} \end{aligned}$$

where $\theta$ is the angle of incidence on the tilted surface and $\theta _z$ is the solar zenith angle.

The angle of incidence is calculated as:

8 $$\begin{aligned} \begin{aligned} \cos \theta ={}&\sin \delta \sin \phi \cos \beta - \sin \delta \cos \phi \sin \beta \cos \gamma \\&+ \cos \delta \cos \phi \cos \beta \cos \omega + \cos \delta \sin \phi \sin \beta \cos \gamma \cos \omega \\&+ \cos \delta \sin \beta \sin \gamma \sin \omega \end{aligned} \end{aligned}$$

Using the isotropic sky model, the diffuse transposition factor is:

9 $$\begin{aligned} R_d = \frac{1 + \cos \beta }{2} \end{aligned}$$

The ground-reflected transposition factor is:

10 $$\begin{aligned} R_r = \frac{1 - \cos \beta }{2} \end{aligned}$$

### PV module electrical modeling

#### Single-diode model

The electrical behavior of a PV cell is accurately represented by the single-diode equivalent circuit model. The current-voltage (I-V) relationship is described by:

11 $$\begin{aligned} I = I_{ph} - I_0 \left[ \exp \left( \frac{V + IR_s}{n_s a V_t}\right) - 1\right] - \frac{V + IR_s}{R_{sh}} \end{aligned}$$

#### Parameter extraction

The photogenerated current varies with irradiance and temperature:

12 $$\begin{aligned} I_{ph} = \left[ I_{sc,ref} + K_i(T_c - T_{ref})\right] \frac{G_T}{G_{ref}} \end{aligned}$$

where $I_{sc,ref}$ is the short-circuit current at reference conditions (STC: $G_{ref} = 1000$ W/m², $T_{ref} = 298.15$ K), and $K_i$ is the temperature coefficient of short-circuit current (A/K).

The reverse saturation current varies with temperature:

13 $$\begin{aligned} I_0 = I_{0,ref} \left( \frac{T_c}{T_{ref}}\right) ^3 \exp \left[ \frac{qE_g}{ak}\left( \frac{1}{T_{ref}} - \frac{1}{T_c}\right) \right] \end{aligned}$$

where $E_g \approx 1.12$ eV is the bandgap energy for silicon.

The reference saturation current is determined from open-circuit conditions:

14 $$\begin{aligned} I_{0,ref} = \frac{I_{sc,ref}}{\exp \left( \frac{V_{oc,ref}}{n_s a V_{t,ref}}\right) - 1} \end{aligned}$$

#### Cell temperature model

The PV cell operating temperature significantly affects module performance. The cell temperature is estimated using the NOCT (Nominal Operating Cell Temperature) model:

15 $$\begin{aligned} T_c = T_a + \left( \frac{NOCT - 20}{800}\right) G_T \end{aligned}$$

where $T_a$ is ambient temperature ($^\circ$C), NOCT is typically 45-47$^\circ$C for crystalline silicon modules, and $G_T$ is in-plane irradiance ($\hbox {W/m}^{2}$).

A more accurate temperature model accounting for wind speed is:

16 $$\begin{aligned} T_c = T_a + \frac{G_T}{U_0 + U_1 \cdot v_w} \left( 1 - \frac{\eta _{module}}{\tau \alpha }\right) \end{aligned}$$

where $U_0$ and $U_1$ are heat transfer coefficients (typically $U_0 = 25$ W/m²K, $U_1 = 6.84$ W·s/m³K), $v_w$ is wind speed (m/s), $\eta _{module}$ is module efficiency, and $\tau \alpha$ is the transmittance-absorptance product (typically 0.9).

#### Maximum power point

The maximum power point (MPP) is determined by solving:

17 $$\begin{aligned} \frac{dP}{dV} = \frac{d(IV)}{dV} = I + V\frac{dI}{dV} = 0 \end{aligned}$$

The maximum power output considering temperature effects is approximated as:

18 $$\begin{aligned} P_{mp} = P_{mp,ref} \frac{G_T}{G_{ref}} \left[ 1 + \gamma (T_c - T_{ref})\right] \end{aligned}$$

where $P_{mp,ref}$ is rated power at STC and $\gamma$ is the temperature coefficient of power (typically -0.35% to -0.45%/$^\circ$C for c-Si modules).

### PV array configuration

For an array with $N_s$ modules in series per string and $N_p$ parallel strings, the array output characteristics are:

19 $$\begin{aligned} V_{array}&= N_s \cdot V_{module} \end{aligned}$$

20 $$\begin{aligned} I_{array}&= N_p \cdot I_{module} \end{aligned}$$

21 $$\begin{aligned} P_{array}&= N_s \cdot N_p \cdot P_{module} \cdot \eta _{mismatch} \end{aligned}$$

where $\eta _{mismatch}$ accounts for mismatch losses between modules (typically 0.98-0.99).

The total number of modules is:

22 $$\begin{aligned} N_{total} = N_s \times N_p = \frac{P_{array,rated}}{P_{module,rated}} \end{aligned}$$

### DC system losses

#### DC cable losses

The DC cable power loss is calculated as:

23 $$\begin{aligned} P_{cable,DC} = I_{DC}^2 \cdot R_{cable,DC} \end{aligned}$$

The cable resistance depends on conductor material, cross-sectional area, length, and temperature:

24 $$\begin{aligned} R_{cable,DC} = \frac{\rho _c \cdot L_{cable}}{A_{cable}} \cdot [1 + \alpha _c(T_{cable} - 20)] \end{aligned}$$

where $\rho _c$ is the electrical resistivity ($1.68\times 10^{-8}\,\Omega \cdot \text {m}$ for copper and $2.82\times 10^{-8}\,\Omega \cdot \text {m}$ for aluminum), $L_{cable}$ denotes the total cable length (m), $A_{cable}$ represents the cable cross-sectional area (m$^{2}$), and $\alpha _c$ is the temperature coefficient of resistance ($0.00393~^\circ \text {C}^{-1}$ for copper).

The relative DC cable loss is typically maintained below 2%:

25 $$\begin{aligned} \eta _{cable,DC} = 1 - \frac{P_{cable,DC}}{P_{DC}} \ge 0.98 \end{aligned}$$

#### Combiner box losses

Combiner box losses include contact resistance, fuse losses, and surge protection device (SPD) losses:

26 $$\begin{aligned} P_{combiner} = n_{strings} \cdot I_{string}^2 \cdot (R_{contact} + R_{fuse}) \end{aligned}$$

Typical combiner box efficiency is 99.5-99.8%.

### Inverter modeling

#### Inverter efficiency curve

The inverter efficiency varies with loading level and is modeled using a quadratic loss model:

27 $$\begin{aligned} \eta _{inv}(P_{in}) = \frac{P_{out}}{P_{in}} = \frac{P_{in} - P_{loss}}{P_{in}} \end{aligned}$$

The power loss comprises standby losses, linear losses, and quadratic losses:

28 $$\begin{aligned} P_{loss} = P_0 + k_1 P_{in} + k_2 P_{in}^2 \end{aligned}$$

where $P_0$ is standby/no-load loss, $k_1$ represents linear losses (switching losses), and $k_2$ represents quadratic losses (conduction losses).

Alternatively, the efficiency can be expressed as:

29 $$\begin{aligned} \eta _{inv} = \frac{p}{p + k_0 + k_1 p + k_2 p^2} \end{aligned}$$

where $p = P_{in}/P_{inv,rated}$ is the normalized input power.

The European weighted efficiency, commonly used for performance comparison, is:

30 $$\begin{aligned} \eta _{EU} = 0.03\eta _{5\%} + 0.06\eta _{10\%} + 0.13\eta _{20\%} + 0.10\eta _{30\%} + 0.48\eta _{50\%} + 0.20\eta _{100\%} \end{aligned}$$

#### MPPT efficiency

The Maximum Power Point Tracking (MPPT) efficiency accounts for the inverter’s ability to track the true MPP:

31 $$\begin{aligned} \eta _{MPPT} = \frac{E_{tracked}}{E_{MPP,ideal}} = \frac{\int P_{tracked}(t) dt}{\int P_{MPP}(t) dt} \end{aligned}$$

Modern inverters achieve MPPT efficiency of 99.0-99.9%.

#### DC/AC sizing ratio

The DC/AC ratio (also called inverter loading ratio or ILR) is defined as:

32 $$\begin{aligned} R_{DC/AC} = \frac{P_{PV,rated}}{P_{inv,rated}} \end{aligned}$$

Optimal DC/AC ratios typically range from 1.1 to 1.4 depending on solar resource, electricity pricing, and component costs. Higher ratios increase energy capture during low-irradiance periods but result in clipping losses during peak irradiance.

The clipping loss is:

33 $$\begin{aligned} E_{clip} = \int _0^{8760} \max (0, P_{DC}(t) - P_{inv,rated}) dt \end{aligned}$$

### AC system and grid integration

#### AC cable losses

Three-phase AC cable losses are calculated as:

34 $$\begin{aligned} P_{cable,AC} = 3 I_{AC}^2 R_{cable,AC} = \frac{P_{AC}^2 R_{cable,AC}}{V_{LL}^2 \cos ^2\phi } \end{aligned}$$

where $V_{LL}$ is line-to-line voltage and $\cos \phi$ is power factor.

#### Transformer losses

Step-up transformer losses comprise no-load (core) losses and load (copper) losses:

35 $$\begin{aligned} P_{transformer} = P_{NL} + P_{LL} \left( \frac{S}{S_{rated}}\right) ^2 \end{aligned}$$

where $P_{NL}$ is no-load loss, $P_{LL}$ is full-load loss, *S* is actual apparent power, and $S_{rated}$ is transformer rating.

Transformer efficiency is:

36 $$\begin{aligned} \eta _{transformer} = \frac{P_{out}}{P_{out} + P_{NL} + P_{LL}(P_{out}/S_{rated})^2} \end{aligned}$$

### Overall system model

The net AC energy delivered to the grid is:

37 $$\begin{aligned} \begin{aligned} E_{AC} = \int _{0}^{8760}&P_{PV}(t)\cdot \eta _{cable,DC}\cdot \eta _{combiner}\cdot \eta _{inv}(t) \\&\cdot \eta _{MPPT}\cdot \eta _{cable,AC}\cdot \eta _{transformer}\cdot (1-L_{aux}) \, dt \end{aligned} \end{aligned}$$

where $L_{aux}$ accounts for auxiliary consumption (typically 0.5-1.5% of generation).

The overall system efficiency is:

38 $$\begin{aligned} \eta _{system} = \frac{E_{AC}}{H_T \cdot A_{array}} \end{aligned}$$

where $H_T$ is total in-plane irradiation and $A_{array}$ is array area.

## Proposed CLF-GWO algorithm

### Standard Grey wolf optimizer

The Grey wolf optimizer (GWO) is inspired by the leadership hierarchy and hunting mechanism of grey wolves (*Canis lupus*). The social hierarchy consists of four levels: the Alpha ($\alpha$), which is the leader wolf responsible for decision-making and represents the best solution found so far in the optimization context; the Beta ($\beta$), which serves as the second-in-command that assists the alpha and represents the second-best solution; the Delta ($\delta$), comprising subordinate wolves including scouts, sentinels, elders, hunters, and caretakers, which represents the third-best solution; and the Omega ($\omega$), the lowest-ranking wolves that submit to all other wolves and represent the remaining candidate solutions. The hunting process involves three main phases: searching for prey (exploration), encircling prey, and attacking prey (exploitation).

#### Encircling prey

The encircling behavior is mathematically modeled as:

39 $$\begin{aligned} \vec {D}&= |\vec {C} \cdot \vec {X}_p(t) - \vec {X}(t)| \end{aligned}$$

40 $$\begin{aligned} \vec {X}(t+1)&= \vec {X}_p(t) - \vec {A} \cdot \vec {D} \end{aligned}$$

where *t* indicates current iteration, $\vec {X}_p$ is position vector of prey (best solution), $\vec {X}$ is position vector of a grey wolf, and $\vec {A}$ and $\vec {C}$ are coefficient vectors calculated as:

41 $$\begin{aligned} \vec {A}&= 2\vec {a} \cdot \vec {r}_1 - \vec {a} \end{aligned}$$

42 $$\begin{aligned} \vec {C}&= 2 \cdot \vec {r}_2 \end{aligned}$$

where $\vec {r}_1$ and $\vec {r}_2$ are random vectors in [0,1], and $\vec {a}$ is linearly decreased from 2 to 0 over iterations:

43 $$\begin{aligned} \vec {a} = 2 - t \cdot \frac{2}{T_{max}} \end{aligned}$$

#### Hunting (position update)

Since the exact location of prey (global optimum) is unknown, alpha, beta, and delta are assumed to have better knowledge of potential prey location. The position update considers all three leaders:

44 $$\begin{aligned} \vec {D}_\alpha&= |\vec {C}_1 \cdot \vec {X}_\alpha - \vec {X}| \end{aligned}$$

45 $$\begin{aligned} \vec {D}_\beta&= |\vec {C}_2 \cdot \vec {X}_\beta - \vec {X}| \end{aligned}$$

46 $$\begin{aligned} \vec {D}_\delta&= |\vec {C}_3 \cdot \vec {X}_\delta - \vec {X}| \end{aligned}$$

47 $$\begin{aligned} \vec {X}_1&= \vec {X}_\alpha - \vec {A}_1 \cdot \vec {D}_\alpha \end{aligned}$$

48 $$\begin{aligned} \vec {X}_2&= \vec {X}_\beta - \vec {A}_2 \cdot \vec {D}_\beta \end{aligned}$$

49 $$\begin{aligned} \vec {X}_3&= \vec {X}_\delta - \vec {A}_3 \cdot \vec {D}_\delta \end{aligned}$$

50 $$\begin{aligned} \vec {X}(t+1)&= \frac{\vec {X}_1 + \vec {X}_2 + \vec {X}_3}{3} \end{aligned}$$

#### Exploration and exploitation

The GWO algorithm balances exploration and exploitation through the $|\vec {A}|$ value:

- When $|\vec {A}| > 1$: Wolves diverge from prey (exploration)
- When $|\vec {A}| < 1$: Wolves converge toward prey (exploitation)

### Chaotic map integration

Chaotic sequences exhibit properties of ergodicity, non-periodicity, and sensitivity to initial conditions, making them effective for enhancing optimization algorithm performance. The proposed CLF-GWO integrates two chaotic maps:

#### Logistic chaotic map

The logistic map is defined as:

51 $$\begin{aligned} z_{k+1} = \mu \cdot z_k \cdot (1 - z_k), \quad z_k \in (0,1), \quad \mu = 4 \end{aligned}$$

The bifurcation parameter $\mu = 4$ ensures fully chaotic behavior with ergodic coverage of the interval (0,1).

#### Tent chaotic map

The tent map provides different chaotic characteristics:

52 $$\begin{aligned} z_{k+1} = {\left\{ \begin{array}{ll} \frac{z_k}{0.7} & \text {if } z_k < 0.7 \\ \frac{10(1-z_k)}{3} & \text {if } z_k \ge 0.7 \end{array}\right. } \end{aligned}$$

#### Chaotic initialization

Population initialization using chaotic sequences improves initial diversity:

53 $$\begin{aligned} X_{i,j}^{(0)} = X_j^{min} + z_i^{(j)} \cdot (X_j^{max} - X_j^{min}) \end{aligned}$$

where $z_i^{(j)}$ is generated using the logistic map with different initial seeds for each dimension.

#### Chaotic parameter adaptation

The convergence parameter $\vec {a}$ is adaptively controlled using chaotic sequences:

54 $$\begin{aligned} a(t) = 2 \cdot \left( 1 - \frac{t}{T_{max}}\right) ^{c_1} \cdot \left( 1 + c_2 \cdot z_t^{tent}\right) \end{aligned}$$

where $c_1 = 2$ controls non-linear decrease, $c_2 = 0.3$ scales chaotic perturbation, and $z_t^{tent}$ is generated by tent map.

### Lévy flight integration

Lévy flight enables efficient exploration through heavy-tailed step length distribution. The step length follows an inverse power-law distribution:

55 $$\begin{aligned} L(s) \sim |s|^{-\beta }, \quad 1 < \beta \le 3 \end{aligned}$$

#### Mantegna’s Algorithm

Lévy-distributed random numbers are generated using Mantegna’s algorithm:

56 $$\begin{aligned} s = \frac{u}{|v|^{1/\beta }} \end{aligned}$$

where $u \sim N(0, \sigma _u^2)$ and $v \sim N(0, 1)$ are Gaussian-distributed random variables, with:

57 $$\begin{aligned} \sigma _u = \left[ \frac{\Gamma (1+\beta )\sin (\pi \beta /2)}{\Gamma \left( \frac{1+\beta }{2}\right) \beta \cdot 2^{(\beta -1)/2}}\right] ^{1/\beta } \end{aligned}$$

For $\beta = 1.5$, we have $\sigma _u \approx 0.6966$.

#### Lévy flight position update

The Lévy flight-enhanced position update is:

58 $$\begin{aligned} \vec {X}_{Levy}(t+1) = \vec {X}(t) + \alpha _0 \cdot s \cdot (\vec {X}(t) - \vec {X}_{best}) \oplus L(\beta ) \end{aligned}$$

where $\alpha _0$ is a step size scaling factor (typically 0.01), and $\oplus$ denotes entry-wise multiplication.

The probability of applying Lévy flight is adaptively controlled:

59 $$\begin{aligned} p_{Levy}(t) = p_{max} - (p_{max} - p_{min}) \cdot \left( \frac{t}{T_{max}}\right) ^2 \end{aligned}$$

where $$p_{max} = 0.5$$ and $$p_{min} = 0.05$$.

### CLF-GWO algorithm framework

**Fig. 2:**
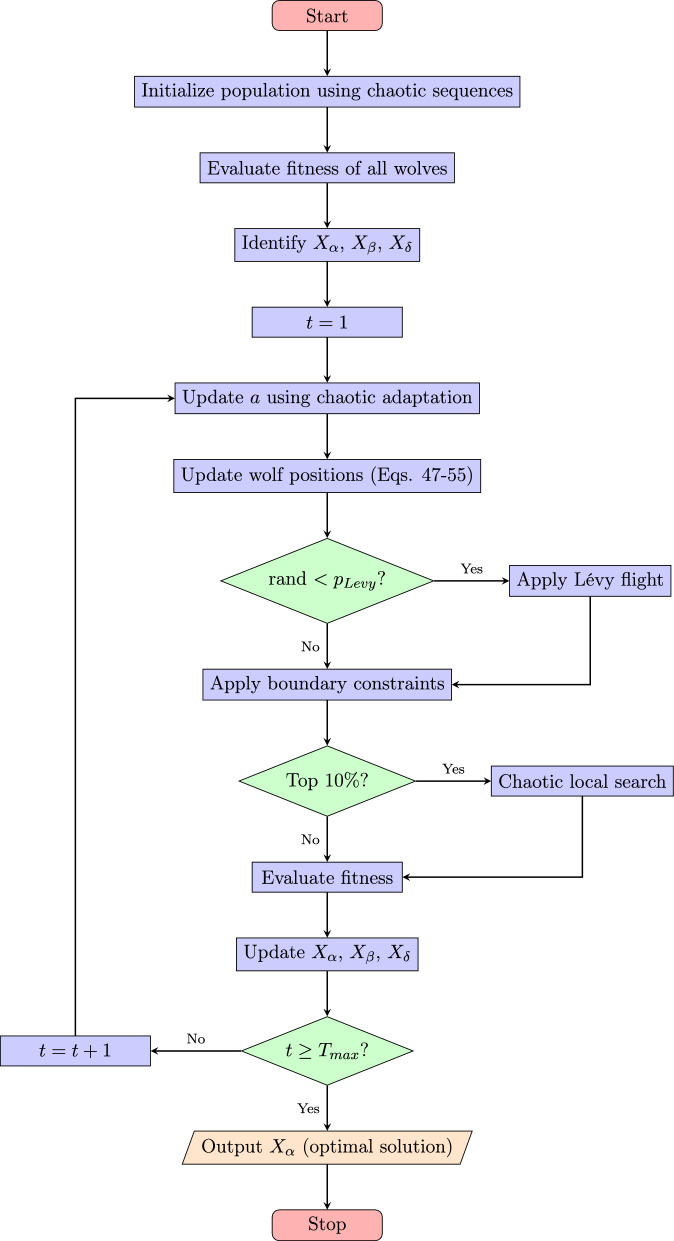
Flowchart of the proposed CLF-GWO algorithm.

The complete CLF-GWO algorithm integrates the above components as shown in the flowchart (Fig. 2). The proposed CLF-GWO algorithm begins with chaotic initialization of the wolf population using logistic map sequences. Initial fitness evaluation identifies the three best solutions ($X_\alpha$, $X_\beta$, $X_\delta$). The iterative optimization loop updates the convergence parameter *a* using chaotic adaptation and modifies wolf positions accordingly. A probabilistic Lévy flight mechanism enhances exploration when triggered. Boundary constraints ensure solution feasibility. Elite solutions (top 10%) undergo chaotic local search for intensified exploitation. After fitness re-evaluation, the hierarchy is updated. This process continues until the maximum iteration count is reached, whereupon the algorithm outputs the optimal solution $X_\alpha$ and terminates (Table 1).

## Multi-objective optimization formulation

### Decision variables

The optimization problem involves determining optimal values for the following decision variables:

**Table 1:** Decision variables and their bounds.

Variable	Description	Min.	Max.	Unit
$P_{PV}$	PV array rated capacity	800	1200	kWp
$P_{inv}$	Inverter rated capacity	700	1100	kVA
$\beta$	Module tilt angle	0	45	Degrees
$\gamma$	Module azimuth angle	-30	30	Degrees
$N_s$	Modules per string	15	30	–
$N_p$	Number of parallel strings	30	80	–

### Objective functions

#### Objective 1: Minimize levelized cost of energy (LCOE)

The LCOE represents the per-unit cost of electricity generation over the project lifetime:

60 $$\begin{aligned} LCOE = \frac{\sum _{n=0}^{N} \frac{C_n}{(1+d)^n}}{\sum _{n=1}^{N} \frac{E_n}{(1+d)^n}} \end{aligned}$$

where $C_n$ is the cost in year *n*, $E_n$ is energy generated in year *n*, *d* is discount rate, and *N* is project lifetime.

For simplified calculation:

61 $$\begin{aligned} LCOE = \frac{C_{capital} \cdot CRF + C_{O[NONSPACE] \& M} + C_{replacement} \cdot PWF}{E_{annual} \cdot (1 - \bar{\delta })^{N/2}} \end{aligned}$$

where:

62 $$\begin{aligned} CRF&= \frac{d(1+d)^N}{(1+d)^N - 1} \end{aligned}$$

63 $$\begin{aligned} PWF&= \frac{1}{(1+d)^{n_{rep}}} \end{aligned}$$

The capital cost breakdown is:

64 $$\begin{aligned} \begin{aligned} C_{capital} ={}&c_{module}\cdot P_{PV} + c_{inv}\cdot P_{inv} + c_{structure}\cdot P_{PV} \\&+ c_{cable}\cdot L_{cable} + c_{transformer} + c_{land}\cdot A_{land} + c_{installation} \end{aligned} \end{aligned}$$

#### Objective 2: Maximize performance ratio (PR)

65 $$\begin{aligned} \max PR = \frac{\sum _{t=1}^{8760} P_{AC}(t) \Delta t}{\sum _{t=1}^{8760} P_{PV,rated} \cdot \frac{G_T(t)}{G_{ref}} \Delta t} \end{aligned}$$

Equivalently, minimize $$(1 - PR)$$:

66 $$\begin{aligned} \min f_2 = 1 - PR \end{aligned}$$

#### Objective 3: Maximize capacity utilization factor (CUF)

67 $$\begin{aligned} \max CUF = \frac{E_{AC,annual}}{P_{PV,rated} \times 8760} \end{aligned}$$

#### Objective 4: Minimize loss of power supply probability (LPSP)

For grid-connected systems with export limitations:

68 $$\begin{aligned} LPSP = \frac{\sum _{t=1}^{8760} \max (0, P_{gen}(t) - P_{grid,max})}{\sum _{t=1}^{8760} P_{gen}(t)} \end{aligned}$$

## Case study: solar power plant in Assam

### Site description

The proposed 1 MWp grid-connected solar power plant is situated in Guwahati, Assam, at coordinates 26.14$^\circ$N latitude and 91.74$^\circ$E longitude, with an elevation of 55 meters above mean sea level. Table 2 presents the detailed site characteristics. Guwahati experiences a subtropical humid climate (Köppen classification Cwa) characterized by warm temperatures averaging 24.2$^\circ$C annually, ranging from 10$^\circ$C in winter to 35$^\circ$C in summer. The site receives moderate solar resources with average global horizontal irradiance (GHI) of 4.35 kWh/m^2^/day, direct normal irradiance (DNI) of 3.82 kWh/m^2^/day, and diffuse horizontal irradiance (DHI) of 2.15 kWh/m^2^/day, while the region experiences approximately 2,190 sunshine hours annually, making it suitable for solar power generation despite monsoon-induced cloudiness. The high relative humidity (78.5%) and substantial annual rainfall (1,722 mm) are characteristic of Assam’s climate, affecting module soiling and temperature-related losses, whereas the low average wind speed (1.8 m/s) limits natural cooling of PV modules. The plant connects to the 33 kV distribution grid operating at 50 Hz frequency, and the available land area of 2.5 hectares adequately accommodates the 1 MWp installation including inter-row spacing for maintenance access and shadow avoidance. These parameters form the basis for system design optimization and energy yield estimation.

**Table 2:** Site specifications for Guwahati, Assam.

Parameter	Value	Unit
Location	Guwahati, Assam	–
Latitude	26.14$^\circ$N	degrees
Longitude	91.74$^\circ$E	degrees
Elevation	55	m above MSL
Climate classification	Subtropical humid (Cwa)	Köppen
Average GHI	4.35	kWh/m²/day
Average DNI	3.82	kWh/m²/day
Average DHI	2.15	kWh/m²/day
Average temperature	24.2	$^\circ$C
Temperature range	10–35	$^\circ$C
Average relative humidity	78.5	%
Annual rainfall	1722	mm
Average wind speed	1.8	m/s
Sunshine hours	2,190	hours/year
Grid voltage	33 kV	–
Grid frequency	50 ± 0.5	Hz
Available land area	2.5	hectares

### Meteorological data

Comprehensive hourly meteorological data covering a period of 36 months, from January 2021 to December 2023, were obtained from the NASA POWER database and subsequently cross-validated with ground-based measurements provided by India Meteorological Department (IMD) stations to ensure data reliability and accuracy. The dataset includes key meteorological parameters relevant to solar energy assessment, namely global horizontal irradiance (GHI), diffuse horizontal irradiance (DHI), direct normal irradiance (DNI), ambient temperature, relative humidity, wind speed and direction, and atmospheric pressure.

**Fig. 3:**
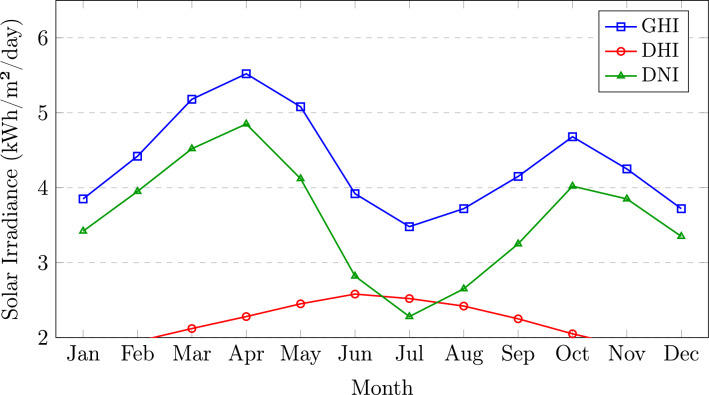
Monthly average daily solar irradiance components for Guwahati, Assam.

Figure 3 illustrates the monthly variation of the solar irradiance components. It is observed that GHI attains its maximum values during the pre-monsoon months of March and April, ranging from 5.18 to 5.52 kWh/m$^{2}$/day, while a pronounced reduction is evident during the monsoon period from June to August, with values decreasing to approximately 3.48–3.92 kWh/m$^{2}$/day. A similar seasonal trend is observed for DNI. In contrast, DHI remains comparatively stable throughout the year, with a marginal increase during the monsoon season attributed to enhanced cloud cover, which increases the proportion of diffuse radiation.

**Fig. 4:**
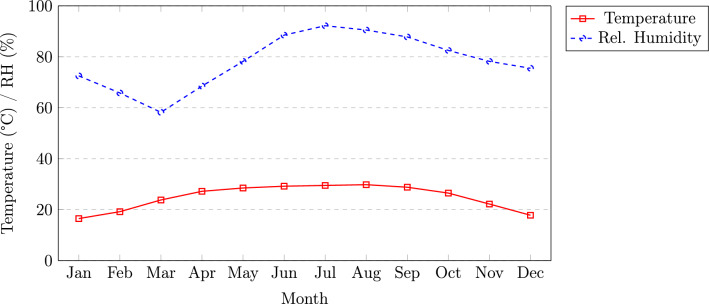
Monthly average temperature and relative humidity for Guwahati, Assam.

The variations in ambient temperature and relative humidity are presented in Fig. 4. The ambient temperature varies from a minimum of approximately 16.5$^\circ$C in January to a maximum of about 29.8$^\circ$C in August. Relative humidity exhibits a strong seasonal dependence, peaking during the monsoon months with values in the range of 88–92%, and declining during the pre-monsoon period to approximately 58–68%. This inverse relationship between temperature and humidity plays a significant role in photovoltaic (PV) module performance, particularly due to temperature-induced efficiency degradation.

**Fig. 5:**
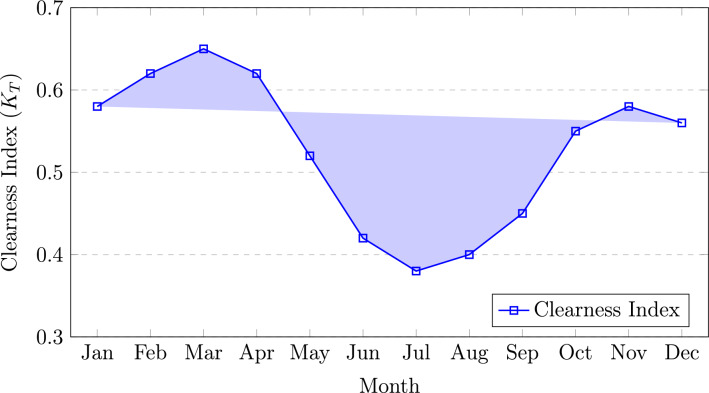
Monthly average clearness index for Guwahati, Assam.

Figure 5 depicts the monthly variation of the clearness index ($K_T$), which serves as an indicator of atmospheric transparency. The clearness index varies from a minimum value of approximately 0.38 during July, corresponding to intense monsoon conditions, to a maximum of about 0.65 in March under clear pre-monsoon skies. These variations in $K_T$ directly influence solar energy availability and are critical for accurate PV system design and performance assessment.

### Component specifications

The proposed 1 MWp solar power plant employs high-efficiency Tier-1 monocrystalline PERC (Passivated Emitter and Rear Cell) photovoltaic modules, the detailed specifications of which are summarized in Table 3. Each module is rated at 545 Wp under standard test conditions (STC), achieving a high conversion efficiency of 21.1%. The key electrical parameters include a maximum power point voltage ($V_{\textrm{mp}}$) of 41.65 V and a corresponding current ($I_{\textrm{mp}}$) of 13.09 A. The open-circuit voltage ($V_{\textrm{oc}}$) and short-circuit current ($I_{\textrm{sc}}$) are 49.65 V and 13.92 A, respectively.

The temperature coefficient of power, specified as $-0.35\%\!/^{\circ }$C, is particularly significant for deployment in Assam’s warm climatic conditions, as elevated operating temperatures lead to a reduction in module output power. The use of a 144 half-cut cell architecture contributes to lower resistive losses, improved current distribution, and enhanced tolerance to partial shading. Furthermore, the modules exhibit a nominal operating cell temperature (NOCT) of $45 \pm 2^{\circ }$C, reflecting realistic field operating conditions. Long-term reliability is ensured through comprehensive warranty coverage, comprising a 12-year product warranty and a 25-year linear power performance guarantee.

**Table 3:** PV module specifications (Monocrystalline PERC).

Parameter	Value	Unit
Manufacturer/Model	Tier-1 / 545 Wp Mono PERC	–
Rated power ($P_{mp}$)	545	Wp
Module efficiency	21.1	%
Voltage at MPP ($V_{mp}$)	41.65	V
Current at MPP ($I_{mp}$)	13.09	A
Open circuit voltage ($V_{oc}$)	49.65	V
Short circuit current ($I_{sc}$)	13.92	A
Temperature coeff. of $P_{mp}$ ($\gamma$)	−0.35	%/$^\circ$C
Temperature coeff. of $V_{oc}$ ($\beta _V$)	−0.27	%/$^\circ$C
Temperature coeff. of $I_{sc}$ ($\alpha _I$)	+0.048	%/$^\circ$C
NOCT	45 ± 2	$^\circ$C
Dimensions (L $\times$ W $\times$ H)	2278 $\times$ 1134 $\times$ 35	mm
Weight	28.6	kg
Cell type	Monocrystalline PERC	–
Number of cells	144 (half-cut)	–
Warranty	12 years product, 25 years linear power	–

**Table 4:** Central inverter specifications.

Parameter	Value	Unit
Rated AC power	1000	kW
Max DC power	1200	kW
Max DC voltage	1500	V
MPPT voltage range	860–1300	V
Number of MPPT	12	–
Max input current per MPPT	26	A
Max efficiency	99.0	%
European efficiency	98.6	%
MPPT efficiency	99.9	%
AC output voltage	600	V
Power factor	0.8 leading - 0.8 lagging	–
THD	< 3	%
Standby consumption	100	W
Operating temperature	−25 to +60	$^\circ$C
Protection class	IP65	–
Cooling	Forced air	–

The specifications of the central inverter employed in the plant are presented in Table 4. The inverter is rated at 1000 kW AC power and is capable of accommodating up to 1200 kW of DC input, thereby allowing a DC/AC ratio of up to 1.2 to maximize annual energy yield. A maximum conversion efficiency of 99.0% and a European weighted efficiency of 98.6% ensure minimal power losses during DC–AC conversion. The inverter supports a wide maximum power point tracking (MPPT) voltage range of 860–1300 V and incorporates 12 independent MPPT channels, providing flexibility in string configuration and ensuring effective power extraction under non-uniform operating conditions. Power quality requirements are satisfied with total harmonic distortion (THD) maintained below 3%. Additionally, the inverter is designed to operate reliably over a broad ambient temperature range from −25to $+60^{\circ }$C, making it well suited for the climatic conditions prevalent in Assam.

### Economic parameters

**Table 5:** Economic parameters for techno-economic analysis.

Parameter	Value	Unit
PV module cost	24,000	₹/kWp
Inverter cost	4500	₹/kVA
Module mounting structure	5500	₹/kWp
DC cabling and accessories	2500	₹/kWp
AC system and transformer	3000	₹/kWp
Land cost (lease)	50,000	₹/acre/year
Civil works and fencing	2000	₹/kWp
SCADA and monitoring	1500	₹/kWp
Engineering and commissioning	1500	₹/kWp
Annual O&M cost	1.2	% of capital
Insurance	0.25	% of capital/year
Discount rate	10	%
Inflation rate	5	%
Debt–equity ratio	70:30	–
Loan interest rate	9.5	%
Loan tenure	15	years
Project lifetime	25	years
Degradation rate (Year 1)	2	%
Degradation rate (Year 2+)	0.55	%/year
Inverter replacement (Year 12)	3500	₹/kVA
Feed-in tariff (APPC)	3.85	₹/kWh
Accelerated depreciation	40	% (Year 1)

Table 5 presents the comprehensive set of economic parameters used for the techno-economic evaluation of the proposed 1 MWp solar power plant in Assam. The capital expenditure (CAPEX) comprises several cost components, including photovoltaic (PV) modules priced at ₹ 24,000/kWp and inverters at ₹ 4500/kVA. The cost of the module mounting structure is estimated at ₹ 5500/kWp, while DC cabling and associated accessories account for ₹ 2500/kWp. The AC-side system, including the transformer, is valued at ₹ 3000/kWp. Additional capital costs include civil works and fencing (₹ 2000/kWp), the SCADA monitoring system (₹ 1500/kWp), and engineering, procurement, and commissioning charges amounting to ₹ 1500/kWp. Land is assumed to be procured on a lease basis at a rate of ₹ 50,000 per acre per year, thereby reducing the initial capital investment. The operational expenditure (OPEX) parameters include annual operation and maintenance (O&M) costs estimated at 1.2% of the total capital cost, along with insurance expenses corresponding to 0.25% of the capital cost per annum. The financial assumptions adopted in the analysis consider a discount rate of 10% and an inflation rate of 5%. A debt–equity ratio of 70:30 is assumed, with the debt portion financed at an interest rate of 9.5% over a loan tenure of 15 years. The overall project lifetime is taken as 25 years.

Module performance degradation is incorporated by assuming a degradation rate of 2% during the first year of operation, followed by a gradual decline of 0.55% per year over the remaining project life. Inverter replacement is scheduled in the 12th year of operation, with a replacement cost of ₹ 3,500/kVA. Revenue generation is evaluated based on a feed-in tariff linked to the Average Power Purchase Cost (APPC), assumed to be ₹ 3.85/kWh. In addition, the availability of accelerated depreciation benefits, with 40% depreciation claimed in the first year, provides significant tax advantages. This incentive plays a crucial role in improving the overall financial viability of the project under the prevailing Indian renewable energy policy framework.

## Results and discussion

### Algorithm configuration

Table 6 presents the CLF–GWO algorithm parameter settings determined through preliminary sensitivity studies. The population size is set to 50 wolves with a maximum of 500 iterations, providing an adequate balance between exploration and exploitation. The logistic map parameter is fixed at $\mu = 4.0$, ensuring fully chaotic behavior and enhanced population diversity. The Lévy flight index is selected as $\beta = 1.5$ to control the heavy-tailed step-length distribution, while the Lévy step scaling factor $\alpha _{0} = 0.01$ regulates the jump magnitude. The adaptive Lévy probability varies from 0.5 during the early exploration phase to 0.05 in the late exploitation stage. The chaotic adaptation parameters $c_{1} = 2.0$ and $c_{2} = 0.3$ govern the non-linear convergence behavior of the algorithm. The optimization weights prioritize levelized cost of energy (LCOE) minimization with $w_{1} = 0.35$, while maintaining a balanced emphasis on performance ratio ($w_{2} = 0.25$), capacity utilization factor (CUF) ($w_{3} = 0.25$), and reliability ($w_{4} = 0.15$). A total of 50 independent runs are performed to ensure the statistical validity and robustness of the results.

**Table 6:** CLF-GWO algorithm parameter settings.

Parameter	Value
Population size (*N*)	50
Maximum iterations ($T_{max}$)	500
Logistic map parameter ($\mu$)	4.0
Lévy index ($\beta$)	1.5
Lévy step scaling ($\alpha _0$)	0.01
$p_{Levy,max}$	0.5
$p_{Levy,min}$	0.05
Chaotic adaptation parameters ($c_1$, $c_2$)	2.0, 0.3
Local search scaling ($\sigma _0$)	0.1
Number of independent runs	50
Optimization weights ($w_1$, $w_2$, $w_3$, $w_4$)	0.35, 0.25, 0.25, 0.15

### Convergence analysis

**Fig. 6:**
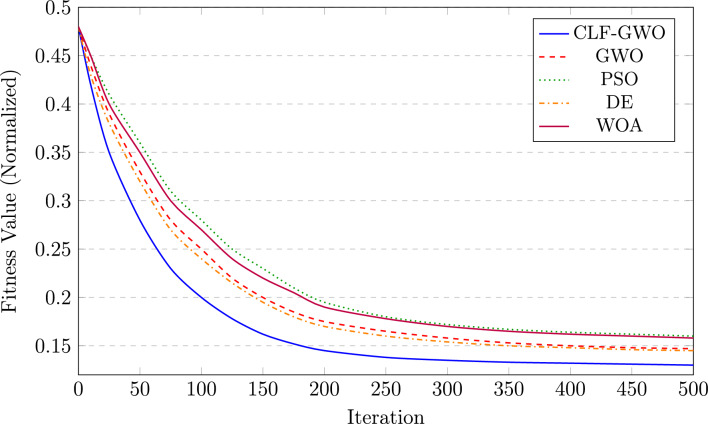
Convergence comparison of optimization algorithms for Assam solar plant.

Figure 6 illustrates the convergence characteristics of five optimization algorithms over 500 iterations. The proposed CLF–GWO algorithm, represented by a blue solid line, exhibits superior convergence behavior by attaining a near-optimal fitness value of 0.130 significantly faster than the other algorithms. In comparison, the standard GWO (red dashed line) converges to a fitness value of 0.147, while the differential evolution (DE) algorithm (orange dash-dotted line) reaches 0.145. Particle swarm optimization (PSO), shown by a green dotted line, and the whale optimization algorithm (WOA), represented by a purple solid line, demonstrate relatively slower convergence, achieving final fitness values of 0.160 and 0.158, respectively. The CLF–GWO algorithm achieves approximately 90% of its final optimization within about 150 iterations, whereas the standard GWO requires more than 250 iterations to reach a comparable level of convergence. The improved initial convergence performance is attributed to chaotic initialization, which enhances population diversity and results in a lower starting fitness value around iteration 25, while the Lévy flight mechanism facilitates effective escape from local optima during iterations 50–150. Furthermore, the presence of a stable convergence plateau beyond iteration 300 indicates robust solution quality without evidence of premature stagnation.

### Optimal system configuration

**Table 7:** Optimal system configuration comparison.

Parameter	CLF-GWO	GWO	PSO	DE	Conv.
PV capacity (kWp)	1,089.0	1072.5	1,098.2	1080.5	1000.0
Inverter capacity (kVA)	900	900	920	900	1000
DC/AC ratio	1.21	1.19	1.19	1.20	1.00
Tilt angle ($^\circ$)	23.5	24.2	23.8	24.0	26.0
Azimuth angle ($^\circ$)	-3.2	-2.5	-4.0	-2.8	0.0
Modules per string	24	24	24	24	23
Number of strings	84	82	84	83	80
Total modules	2016	1968	2016	1992	1,840
Array area ($\hbox {m}^{2}$)	5207	5083	5207	5145	4753

Table 7 compares the optimal system configurations obtained using different optimization approaches. The CLF–GWO algorithm results in a photovoltaic (PV) capacity of 1089 kWp coupled with a 900 kVA inverter, leading to a DC/AC ratio of 1.21, which is higher than the conventional ratio of 1.0. This deliberate DC oversizing strategy enhances energy capture during low-irradiance conditions while incurring only marginal clipping losses at peak generation periods. The optimal tilt angle identified by CLF–GWO is $23.5^{\circ }$, which is lower than the conventional latitude-based tilt of approximately $26^{\circ }$, thereby improving the utilization of diffuse solar radiation during Assam’s predominantly cloudy monsoon months. In addition, a slight westward azimuth orientation of $-3.2^{\circ }$ shifts the peak power generation toward afternoon hours, aligning more closely with typical grid demand profiles. The resulting configuration employs 24 modules per string with 84 parallel strings, yielding a total of 2,016 PV modules and an effective array area of 5207 m$^{2}$. Although this array area is approximately 9.5% larger than that of a conventional design, it delivers a substantially higher annual energy yield.

### Performance metrics

**Table 8:** Annual performance metrics comparison.

Metric	CLF-GWO	GWO	PSO	DE	Conv.
Energy yield (MWh/year)	1,542.3	1,508.5	1,495.2	1,512.8	1,350.2
Specific yield (kWh/kWp)	1,416.2	1,406.5	1,361.5	1,400.1	1,350.2
Performance ratio (%)	79.8	78.5	77.2	78.2	71.4
CUF (%)	17.6	17.2	16.8	17.1	13.7
System efficiency (%)	16.8	16.5	16.2	16.4	15.1
LCOE (₹/kWh)	3.89	4.02	4.15	4.05	5.10
Clipping loss (%)	1.8	1.5	1.6	1.6	0.0

Table 8 presents a comprehensive comparison of annual performance metrics for the considered optimization approaches. The CLF–GWO optimized system achieves an annual energy yield of 1,542.3 MWh with a specific yield of 1,416.2 kWh/kWp, outperforming all benchmark algorithms. The corresponding performance ratio of 79.8% reflects world-class performance under subtropical humid climatic conditions and represents a substantial improvement over the conventional design, which attains a performance ratio of only 71.4%. The capacity utilization factor (CUF) of 17.6% is significantly higher than the conventional value of 13.7%, indicating more effective utilization of the available solar resource. System efficiency improves to 16.8% compared to 15.1% for the conventional configuration. Most notably, the levelized cost of energy (LCOE) is reduced to ₹ 3.89/kWh from ₹ 5.10/kWh, corresponding to a reduction of 23.7%, thereby enhancing the economic competitiveness of the project. The observed clipping loss of 1.8% is an acceptable trade-off associated with the DC/AC ratio of 1.21, as the additional energy harvested during low-irradiance periods significantly outweighs the losses incurred during peak generation hours.

### Monthly energy generation

**Fig. 7:**
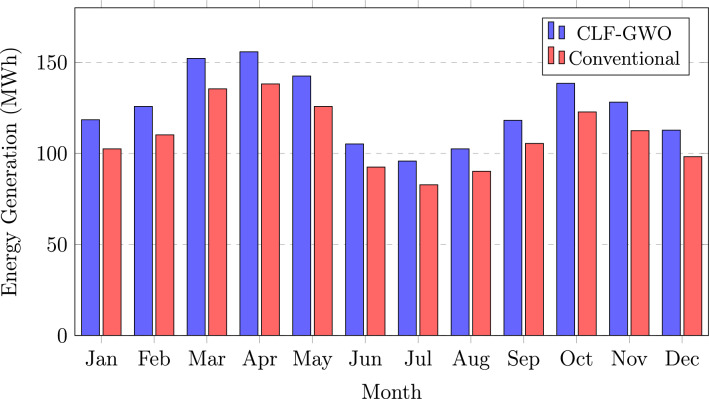
Monthly energy generation comparison for Guwahati, Assam.

Figure 7 illustrates the monthly energy generation comparison between the CLF–GWO optimized system and the conventional design. The CLF–GWO configuration, represented by blue bars, consistently outperforms the conventional system, shown by red bars, across all months of the year. Peak energy generation is observed during the pre-monsoon months of March and April, with monthly outputs ranging from 152.2 to 155.8 MWh, corresponding to higher solar irradiance levels and relatively moderate ambient temperatures. A pronounced reduction in energy generation is evident during the monsoon period from June to August, with monthly values between 95.8 and 105.2 MWh, primarily due to extensive cloud cover and increased atmospheric scattering leading to higher diffuse radiation. Despite these adverse conditions, the CLF–GWO optimized system maintains a consistent performance advantage of approximately 15–16% even during low-generation months, highlighting the effectiveness of the optimized tilt angle in harnessing diffuse solar radiation. The post-monsoon recovery phase is characterized by a notable increase in generation, with October achieving approximately 138.5 MWh as a result of clearer skies and reduced relative humidity. Overall, the annual energy generation improvement of 192.1 MWh, corresponding to a 14.2% increase, directly contributes to higher revenue generation and significantly improved project economics.

### Monthly performance ratio

**Fig. 8:**
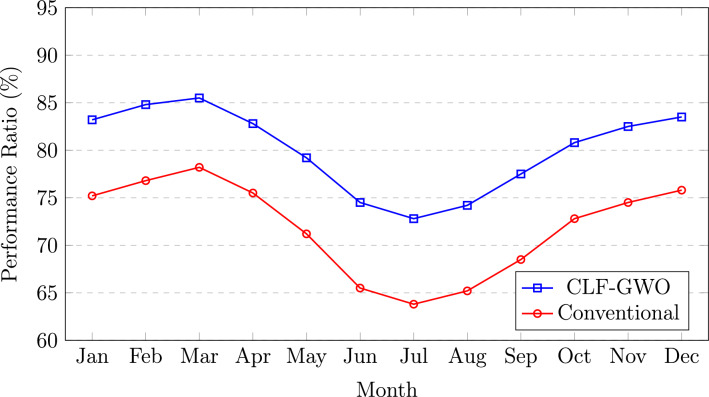
Monthly performance ratio comparison.

Figure 8 presents the monthly variation of the performance ratio (PR) over the annual cycle. The CLF–GWO optimized system, depicted by a blue line with square markers, achieves its highest PR values during the late winter and pre-monsoon months of February and March, ranging from 84.8% to 85.5%, when clear sky conditions and moderate ambient temperatures offer favorable operating environments. A noticeable reduction in PR is observed during the monsoon season, with a minimum value of 72.8% in July, primarily attributed to a higher fraction of diffuse radiation, increased atmospheric humidity, and enhanced module soiling effects. The conventional system, represented by a red line with circular markers, follows a similar seasonal trend but consistently exhibits lower PR values, varying from 63.8% in July to a maximum of 78.2% in March. The performance disparity between the two configurations becomes more pronounced during the monsoon months, where the CLF–GWO optimized system maintains an advantage of approximately 9 percentage points. This observation highlights that the benefits of optimization are most significant under adverse climatic conditions, thereby enhancing system resilience to seasonal variability. Furthermore, the recovery in PR during the winter months of November and December, with values in the range of 82.5–83.5%, confirms the effectiveness of the optimized system design in accommodating the characteristic climatic patterns of Assam.

### Statistical analysis

**Table 9:** Statistical comparison over 50 independent runs.

Algorithm	Best	Mean	Worst	Std	Median	Time (s)
CLF-GWO	0.130	0.133	0.142	0.0035	0.132	85.2
GWO	0.147	0.154	0.168	0.0062	0.153	72.5
PSO	0.160	0.168	0.185	0.0078	0.167	65.8
DE	0.145	0.152	0.165	0.0058	0.151	78.2
WOA	0.158	0.165	0.182	0.0072	0.164	70.5

Table 9 presents a statistical comparison of the optimization results obtained from 50 independent runs, thereby ensuring the reliability and robustness of the analysis. The proposed CLF–GWO algorithm attains the best fitness value of 0.130, with a mean fitness of 0.133 and the lowest standard deviation of 0.0035, indicating highly consistent convergence toward high-quality solutions. The narrow spread between the best (0.130) and worst (0.142) fitness values further confirms the robustness and stability of the proposed approach. In comparison, the standard GWO algorithm exhibits a higher mean fitness value of 0.154 and a larger standard deviation of 0.0062, reflecting reduced solution quality and increased variability. PSO demonstrates the weakest performance, with a mean fitness of 0.168 and a standard deviation of 0.0078, while DE shows relatively competitive performance with a mean fitness of 0.152, albeit with higher variability than CLF–GWO. The proposed CLF–GWO algorithm requires an average computation time of 85.2 s, which is marginally higher than that of the benchmark algorithms due to the additional computational overhead associated with chaotic sequence generation and Lévy flight operations; however, this increase is justified by the significant improvement in solution quality. Furthermore, Wilcoxon signed-rank tests confirm that the performance improvements achieved by CLF–GWO are statistically significant, with *p*-values less than 0.01 when compared against all benchmark algorithms, thereby validating the results at a 99% confidence level.

### Loss analysis

**Fig. 9:**
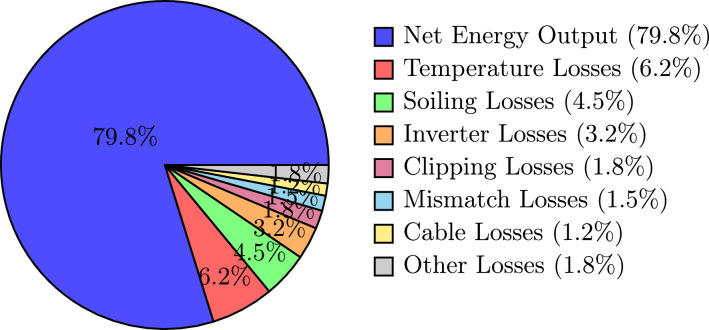
Annual energy loss breakdown for CLF-GWO optimized system.

Figure 9 presents the annual energy loss distribution of the CLF–GWO optimized system using a pie-chart representation. The net energy output accounts for 79.8% of the theoretical maximum energy yield, which is consistent with the achieved system performance ratio. Temperature-related losses constitute the largest share at 6.2%, which is largely unavoidable under Assam’s warm climatic conditions, where photovoltaic cell temperatures frequently exceed 50$^\circ$C during summer afternoons. Soiling losses, amounting to 4.5%, arise from dust deposition and biological growth promoted by the region’s humid environment, thereby highlighting the importance of regular module cleaning and maintenance practices. Inverter losses contribute 3.2% of the total losses, reflecting inherent DC–AC conversion limitations despite the use of high-efficiency inverters with a European efficiency of 98.6%. Clipping losses of 1.8% occur when the DC power exceeds the inverter rated capacity during peak irradiance periods; however, this is an acceptable compromise associated with the selected DC/AC ratio of 1.21, which enhances the overall annual energy yield. Mismatch losses, estimated at 1.5%, result from variations in module electrical characteristics and partial shading effects. Cable losses are limited to 1.2% through appropriate conductor sizing and layout optimization. The remaining 1.8% of losses are attributed to other factors, including transformer losses, auxiliary power consumption, and grid availability constraints. This detailed loss breakdown validates the accuracy of the system modeling approach and provides insight into key areas for further performance optimization.

## Techno-economic analysis

### Capital cost breakdown

**Table 10:** Detailed capital cost breakdown for a 1 MWp solar power plant.

Component	Quantity	Unit Cost (₹)	Total Cost (₹ Lakhs)
PV modules (545 Wp)	2,016 nos	13,080/module	263.69
Central inverter (1000 kW)	1 no	4,500,000	45.00
Module mounting structure	1,089 kWp	5,500/kWp	59.90
DC cables and connectors	Lump sum	–	27.22
AC cables and switchgear	Lump sum	–	18.50
Step-up transformer (1250 kVA)	1 no	1,250,000	12.50
33 kV transmission line	0.5 km	800,000/km	4.00
SCADA and monitoring	Lump sum	–	16.34
Civil works and fencing	Lump sum	–	21.78
Land development	2.5 ha	–	8.50
Engineering and design	Lump sum	–	12.00
Installation and commissioning	Lump sum	–	18.00
Contingency (5%)	–	–	25.37
Total capital cost			532.80
Specific cost			48.93 ₹/Wp

Table 10 presents the detailed capital cost breakdown for the 1 MWp CLF–GWO optimized solar power plant in Assam. The total capital expenditure amounts to ₹ 532.80 Lakhs, corresponding to a specific investment cost of ₹ 48.93/Wp. Photovoltaic (PV) modules represent the largest cost component, totaling ₹ 263.69 Lakhs for 2,016 modules rated at 545 Wp each, with a unit price of ₹ 13,080 per module. The central inverter with a rated capacity of 1000 kW contributes ₹ 45.00 Lakhs, while the module mounting structure accounts for ₹ 59.90 Lakhs, based on a cost of ₹ 5,500/kWp. The electrical infrastructure includes DC cables and connectors costing ₹ 27.22 Lakhs, AC cables and switchgear amounting to ₹ 18.50 Lakhs, and a 1250 kVA step-up transformer valued at ₹ 12.50 Lakhs. The 0.5 km, 33 kV transmission line required for grid interconnection adds an additional cost of ₹ 4.00 Lakhs. Balance-of-system components comprise SCADA and monitoring systems (₹ 16.34 Lakhs), civil works and fencing (₹ 21.78 Lakhs), and land development activities (₹ 8.50 Lakhs). Soft costs include engineering and design services amounting to ₹ 12.00 Lakhs, along with installation and commissioning expenses of ₹ 18.00 Lakhs. Finally, a contingency provision of 5%, equivalent to ₹ 25.37 Lakhs, is incorporated to account for unforeseen expenditures and potential price escalations during project execution.

### Financial analysis

**Table 11:** Financial performance indicators.

Indicator	CLF-GWO	Conventional
Total capital cost (₹ Lakhs)	532.80	488.50
Annual O&M cost (₹ Lakhs)	6.39	5.86
Annual insurance (₹ Lakhs)	1.33	1.22
Annual revenue (₹ Lakhs) @ ₹3.85/kWh	59.38	51.98
Net present value – NPV (₹ Lakhs)	285.42	198.65
Internal rate of return – IRR (%)	16.8	13.2
Equity IRR (%)	22.5	17.8
Simple payback period (years)	5.8	7.2
Discounted payback period (years)	7.5	9.8
LCOE (₹/kWh)	3.89	5.10
Return on investment – ROI (%)	153.6	140.7
Benefit–cost ratio	1.54	1.41

Table 11 compares the financial performance indicators of the CLF–GWO optimized system with those of the conventional system design. Although the CLF–GWO configuration entails a higher initial capital investment of ₹ 532.80 Lakhs compared to ₹ 488.50 Lakhs for the conventional system, it delivers markedly superior financial performance owing to its enhanced annual energy generation. The annual operation and maintenance (O&M) cost for the CLF–GWO system is estimated at ₹ 6.39 Lakhs, corresponding to 1.2% of the capital cost, while insurance expenses amount to ₹ 1.33 Lakhs per year, equivalent to 0.25% of the capital cost. At a feed-in tariff of ₹ 3.85/kWh, the annual revenue generated by the CLF–GWO optimized plant reaches ₹ 59.38 Lakhs, compared to ₹ 51.98 Lakhs for the conventional design, representing a 14.2% increase that directly reflects the higher energy yield. The net present value (NPV) of the CLF–GWO system is calculated as ₹ 285.42 Lakhs, significantly higher than the ₹ 198.65 Lakhs obtained for the conventional system, corresponding to a 43.7% improvement. The project internal rate of return (IRR) increases to 16.8% from 13.2%, while the equity IRR improves to 22.5% compared to 17.8%, indicating strong financial attractiveness for investors. Furthermore, the simple payback period is reduced from 7.2 years to 5.8 years, and the discounted payback period improves from 9.8 years to 7.5 years. The levelized cost of energy (LCOE) for the CLF–GWO system is ₹ 3.89/kWh, which, when accounting for applicable tax benefits, is effectively competitive with the feed-in tariff of ₹ 3.85/kWh, thereby confirming the project’s economic viability. A benefit–cost ratio of 1.54 further indicates that the project yields a return of ₹ 1.54 for every rupee invested.

### Cash flow analysis

**Fig. 10:**
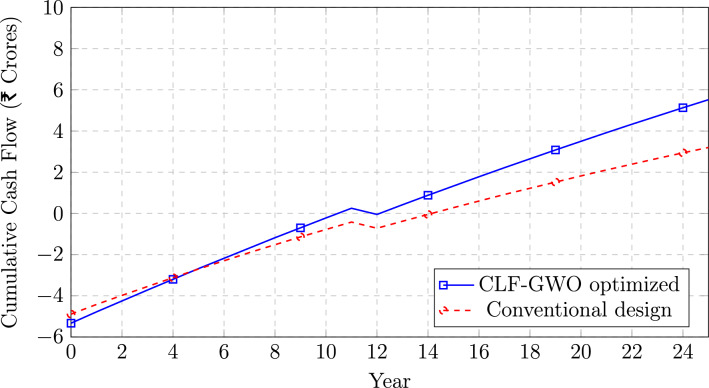
Cumulative discounted cash flow comparison.

Figure 10 illustrates the cumulative discounted cash flow over the 25-year project lifetime for both the CLF–GWO optimized system (blue solid line) and the conventional design (red dashed line). Both projects commence with negative cash flows corresponding to the initial capital investments of approximately ₹ 5.33 Crores for the CLF–GWO system and ₹ 4.89 Crores for the conventional configuration. Owing to higher annual energy generation and revenue, the CLF–GWO optimized system exhibits a steeper cash flow recovery trajectory. The optimized system attains the breakeven point, corresponding to zero cumulative discounted cash flow, at around Year 11, whereas the conventional system reaches breakeven near Year 14, indicating an improvement of nearly three years. A noticeable decline in cumulative cash flow is observed at Year 12 for both systems due to the scheduled inverter replacement cost; however, the CLF–GWO system demonstrates faster recovery and returns to positive cash flow by Year 13. By the end of the project life (Year 25), the CLF–GWO optimized plant achieves a cumulative discounted cash flow of approximately ₹ 5.52 Crores, significantly higher than the ₹ 3.20 Crores realized by the conventional design, corresponding to a 72.5% increase in lifetime returns. The consistently widening gap between the two cash flow curves beyond Year 10 clearly highlights the compounding long-term financial benefits achieved through system optimization.

### Sensitivity analysis

**Fig. 11:**
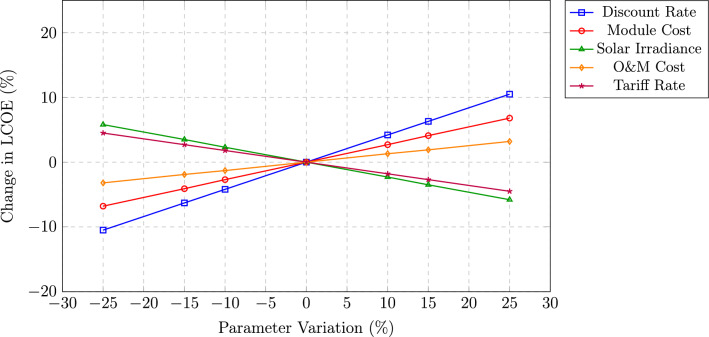
Sensitivity of LCOE to key parameters.

Figure 11 presents a sensitivity analysis illustrating the response of the levelized cost of energy (LCOE) to $\pm 25\%$ variations in key input parameters, represented in the form of a tornado-style diagram. The discount rate, depicted by a blue line with square markers, exhibits the highest sensitivity, where a $\pm 25\%$ variation results in an approximately $\pm 10.5\%$ change in LCOE. This behavior highlights the capital-intensive nature of solar photovoltaic projects, in which financing conditions and the cost of capital exert a dominant influence on levelized energy costs. The PV module cost, represented by a red line with circular markers, shows moderate sensitivity, with a $\pm 25\%$ variation leading to a $\pm 6.8\%$ change in LCOE, underscoring the continued economic benefits associated with declining module prices. Solar irradiance, illustrated by a green line with triangular markers, demonstrates an inverse relationship with LCOE, where a 25% increase in irradiance results in a 5.8% reduction in LCOE due to enhanced annual energy generation. This parameter captures the inherent uncertainty associated with solar resource availability and climatic variability. The operation and maintenance (O&M) cost, shown by an orange line with diamond markers, exhibits the lowest sensitivity, with only a $\pm 3.2\%$ impact on LCOE for a $\pm 25\%$ variation, reflecting the relatively small contribution of operational expenses to the overall lifecycle cost. The tariff rate, indicated by a purple line with star markers, results in a $\pm 4.5\%$ change in LCOE, directly influencing project revenue streams. Notably, the LCOE remains below ₹ 5.00/kWh across all considered parameter variations within the $\pm 25\%$ range, confirming the robustness of the project’s economic viability under uncertainty. The largely symmetric sensitivity trends further indicate near-linear model behavior without pronounced threshold effects.

### Environmental impact

**Table 12:** Environmental impact assessment.

Parameter	CLF-GWO	Conventional
Annual generation (MWh)	1,542.3	1,350.2
Grid emission factor (kg CO$_2$/kWh)	0.82	0.82
Annual CO$_2$ avoided (tonnes)	1,264.7	1,107.2
Lifetime CO$_2$ avoided (tonnes)	28,615	25,048
Equivalent diesel saved (liters/year)	425,800	372,600
Equivalent trees planted	57,468	50,296
Equivalent households powered	428	375
Energy payback time (years)	1.8	2.1
Carbon payback time (years)	2.2	2.5

Table 12 quantifies the environmental benefits achieved by the CLF–GWO optimized system in comparison with the conventional solar PV configuration. The higher annual energy generation of 1,542.3 MWh obtained with the CLF–GWO design, relative to 1,350.2 MWh for the conventional system, directly governs the magnitude of the associated environmental impacts. Based on the Indian grid emission factor of 0.82 kg CO$_2$/kWh, the optimized system achieves an annual CO$_2$ emissions avoidance of 1,264.7 tonnes, compared to 1,107.2 tonnes for the conventional design, corresponding to a 14.2% improvement in emissions reduction. Over the 25-year project lifetime, the cumulative CO$_2$ avoidance amounts to approximately 28,615 tonnes for the CLF–GWO system, as opposed to 25,048 tonnes for the conventional configuration, which is environmentally equivalent to removing nearly 6,200 passenger vehicles from the road each year. In terms of fossil fuel displacement, the optimized plant offsets approximately 425,800 liters of diesel annually, assuming equivalent electricity generation from diesel generators, compared to 372,600 liters displaced by the conventional system. Environmental equivalence indicators further reveal that the CLF–GWO optimized system corresponds to planting about 57,468 trees, whereas the conventional design is equivalent to 50,296 trees, and the optimized plant supplies clean electricity to approximately 428 average Indian households, compared to 375 households served by the conventional system. The energy payback time, defined as the period required for the system to generate energy equal to that consumed during its manufacturing, is reduced to 1.8 years for the CLF–GWO system from 2.1 years for the conventional design, reflecting improved energy yield efficiency. Similarly, the carbon payback time decreases from 2.5 years to 2.2 years. Collectively, these results indicate that more than 92% of the project lifetime contributes net-positive environmental benefits, thereby reinforcing the role of the optimized solar PV system as a genuinely sustainable solution for Assam’s long-term energy transition.

## Conclusions and future research

This study proposed and validated a Chaotic Lévy flight-enhanced Grey wolf optimizer (CLF-GWO) for the optimal design of grid-connected solar photovoltaic (PV) power plants under the subtropical humid climatic conditions of Assam, India. By integrating chaotic population initialization using logistic and tent maps with Lévy flight–based exploration and adaptive parameter control, the proposed framework effectively balances global exploration and local exploitation. Validation using 36 months of high-resolution meteorological data from Guwahati demonstrates that the CLF–GWO algorithm achieves significantly faster and more reliable convergence than conventional metaheuristic techniques. Statistical analysis based on 50 independent runs confirms a best fitness value of 0.130 with a very low standard deviation of 0.0035, and nonparametric Wilcoxon signed-rank tests verify the statistical significance of the improvements at a 99% confidence level.

The optimized system configuration identified by CLF–GWO consists of a 1,089 kWp PV array coupled with a 900 kVA inverter, resulting in an optimal DC/AC ratio of 1.21. The derived tilt angle of $23.5^{\circ }$ and a slight westward azimuth of $-3.2^{\circ }$ are particularly well suited to Assam’s monsoon-dominated climate, enabling enhanced capture of diffuse radiation and improved afternoon energy generation. The optimized plant achieves an annual energy yield of 1,542.3 MWh with a performance ratio of 79.8%, representing improvements of 14.2% and 11.8%, respectively, over a conventional design. The capacity utilization factor increases to 17.6%, while the levelized cost of energy is reduced by 23.7% to ₹ 3.89/kWh. Comprehensive techno-economic analysis demonstrates strong financial viability, with a project internal rate of return of 16.8%, equity IRR of 22.5%, and a simple payback period of 5.8 years. Environmental assessment further highlights annual CO$_2$ emissions avoidance of 1,264.7 tonnes and an energy payback time of only 1.8 years, confirming that more than 92% of the project lifetime delivers net-positive environmental benefits.

Future research should extend the proposed optimization framework to emerging PV technologies and system configurations relevant to northeastern India. In particular, bifacial PV module optimization should be explored to exploit the region’s high diffuse irradiance, which may provide an additional 10–15% energy gain. The integration of single-axis tracking systems could further enhance morning and evening generation profiles. Hybrid solar–wind–hydro systems combined with battery energy storage offer promising solutions for seasonal resource complementarity and grid flexibility. Advanced data-driven approaches, including long short-term memory (LSTM) networks, can be incorporated for improved irradiance forecasting, degradation modeling, and predictive maintenance. Long-term climate change impact assessments considering potential shifts in monsoon patterns over a 25-year horizon would strengthen investment decision-making. Additional research on agrivoltaic systems, floating solar PV on Brahmaputra floodplains, and grid-support functionalities such as virtual synchronous generator control is recommended. Finally, the development of digital twins for real-time monitoring and adaptive optimization could maximize system performance and asset value throughout the operational lifetime.

## Data Availability

The data supporting the findings of this study are openly available in figshare at (https://doi.org/10.6084/m9.figshare.31066891)
